# Decreasing postoperative cognitive deficits after heart surgery: protocol for a randomized controlled trial on cognitive training

**DOI:** 10.1186/s13063-019-3799-0

**Published:** 2019-12-16

**Authors:** Marius Butz, Jasmin El Shazly, Gebhard Sammer, Marlene Tschernatsch, Sabrina Kastaun, Mesut Yenigün, Tobias Braun, Manfred Kaps, Andreas Böning, Ulrike Puvogel, Georg Bachmann, Thomas Mengden, Markus Schönburg, Tibo Gerriets, Martin Juenemann

**Affiliations:** 1 Department of Neurology, Heart and Brain Research Group, University Hospital Giessen and Marburg, Klinikstraße 33, 35385 Giessen, Germany; 2 Department of Cardiac Surgery, Kerckhoff Heart and Thorax Center, Benekestraße 2-8, 61231 Bad Nauheim, Germany; 3 Cognitive Neuroscience at the Centre of Psychiatry, University Giessen, Klinikstraße 36, 35385 Giessen, Germany; 4 Department of Neurology, Community Hospital Friedberg, Ockstädter Straße 3-5, 61169 Friedberg, Germany; 5 Institute of General Practice, Addiction Research and Clinical Epidemiology Unit, Medical Faculty of the Heinrich-Heine University Düsseldorf, Werdener Straße 4, 40227 Düsseldorf, Germany; 6 Department of Cardiovascular Surgery, University Hospital Giessen and Marburg, Rudolf-Buchheim-Straße 7, 35385 Giessen, Germany; 7 Department of Radiology, Kerckhoff Heart and Thorax Center, Benekestraße 2-8, 61231 Bad Nauheim, Germany; 8 Department of Rehabilitation, Kerckhoff Heart and Thorax Center, Ludwigstraße 41, 61231 Bad Nauheim, Germany

**Keywords:** Heart surgery, Postoperative cognitive deficits, Postoperative cerebral microinfarcts, Magnetic resonance imaging, Cognitive training

## Abstract

**Background:**

The occurrence of postoperative cognitive deficits, especially after heart surgery, has been demonstrated in several studies. These deficits can clearly be noticed by the patients and by their close relatives in daily life. Furthermore, postoperative cognitive deficits can decrease quality of life in social functioning and earning capacity. The aim of this study is to investigate whether early postoperative cognitive training can reduce subjective and objective postoperative cognitive deficits.

**Methods:**

The proposed study is a multicenter, two-arm, randomized controlled trial involving 144 elderly patients undergoing elective heart-valve surgery with extracorporeal circulation. Patients will be assigned to either a training group or a control group. The intervention involves paper-and-pencil-based cognitive training, which is conducted for 36 min over a period of 18 days. The training starts about 1 week after surgery and is carried out during the hospitalized rehabilitation phase. The control group will not receive cognitive training or a placebo intervention. A detailed assessment of psychological functions and health-related quality of life prior to surgery at discharge from rehabilitation and 3 and 12 months after discharge will be performed. The primary outcome of this trial is the training effect on objective cognitive functions at discharge from rehabilitation. Secondary outcomes are the training effect on objective and subjective cognitive functions (3 and 12 months after discharge), depression, health-related quality of life, and the impact of perioperative cerebral ischemia on the training effect. Perioperative cerebral ischemia will be measured with postoperative magnetic resonance imaging including diffusion-weighted sequences.

**Discussion:**

Should it be shown that our cognitive training can improve postoperative cognitive deficits and quality of life, one possibility could be to integrate this intervention into early rehabilitation. Furthermore, we hope that the investigation of perioperative ischemia by diffusion-weighted magnetic resonance imaging will improve our understanding of neurobiological factors influencing the course of postoperative cognitive plasticity.

**Trial registration:**

German Clinical Trials Register (DRKS), DRKS00015512. Retrospectively registered on 21 September 2018.

## Background

Neurological complications of cardiac surgery include ischemic and hemorrhagic stroke, seizures, delirium, cerebral hyperperfusion syndrome, cranial and peripheral nerve injuries, and postoperative cognitive decline (POCD) [1]. Amongst these, POCD seems to have the highest incidence, but its frequency strongly depends on the inclusion/exclusion criteria, the follow-up interval and the diagnostic criteria used [2]. There is no uniform definition of POCD, but a widespread criterion for POCD is a decline of 1 standard deviation from preoperative to 3 months postoperative in at least two objectively measured cognitive functions such as verbal memory, attention, cognitive flexibility, language, or visuomotor abilities [2]. Three months after coronary artery bypass grafting (CABG) surgery, POCD occurs in 16% [3] to 23% [4] of patients, and POCD has even been reported in 31% of patients 3 years after undergoing CABG [4]. A longitudinal study assessing the incidence of POCD in CABG surgery reported early improvement in cognitive function within 6 months after surgery that was followed by a later decline and led to a 42% incidence of POCD after 5 years. In this study, early POCD could be identified as an important predictor of long-term cognitive decline [5]. Even though POCD often appears as subclinical, both patients and their relatives report a significant decrease in patients’ cognitive abilities in daily living up to at least 3 months after heart surgery [6]. In addition to POCD, postoperative cognitive improvement (POCI) is also reported, but its frequency seems to be 3–6 times lower than POCD [7].

Several perioperative conditions are discussed as potential pathophysiological mechanisms causing and promoting cerebral injury, such as global and regional hypoperfusion, pronounced temperature, arrhythmia, systemic inflammatory response, hemodilution, anesthesia itself and, particularly, cerebral (micro and macro) embolization followed by ischemia/reperfusion injury and subsequent blood-brain barrier dysfunction [8, 9]. A meta-analysis of randomized controlled trials has shown higher incidence of POCD in CABG with extracorporeal circulation (cardiopulmonary bypass, on-pump) compared to off-pump CABG 3 months after surgery [10]. In this respect, cerebral micro-embolization has been reported to substantially contribute to perioperative neurological complications [11].

Although the pathophysiology of POCD is still the subject of controversial debate, consequences of cognitive deficits are well-described. Aside from a decline in health-related quality of life (HQL) up to 5 years after surgery [12], POCD can result in reduced working and earning capacity with premature retirement, and diminished social functioning resulting in increased social dependency [13]. Cognitive abilities substantially contribute to personality and self-perception; in this light, daily perceived POCD can impose a profound and heavy burden on those concerned. For patients, relatives, and the healthcare system, it is of particular significance that POCD occurs during a highly vulnerable period of life, where cognitive deficits can quickly lead to loss of independence and thus increased need of long-term care [14].

Several studies have shown that patients with mild cognitive impairment (MCI) can benefit from computerized cognitive training [15]. These effects are mainly attributed to cognitive and neurological plasticity (i.e., the ability of the brain to alternate cognitive functions and structural or functional neurophysiological parameters through stimulation). In response to cognitive training, older healthy adults reproducibly present with an increased pattern in the gray and white matter structure [16]. Functional plasticity, on the other hand, shows a mixed pattern of increased and decreased activity in specific brain regions in older healthy adults and consistent increased activity in individuals with MCI, as a result of cognitive training [16]. Furthermore, a functional magnetic resonance imaging (MRI) study reported increased resting-state functional connectivity between the hippocampus and certain brain regions after effective cognitive training in patients with stroke [17]. Cognitive training is also associated with improvements in depression and everyday functioning [18]. To date, only one prospective investigation has addressed the value of cognitive training in patients undergoing cardiac surgery [19]. The authors reported a beneficial effect of memory and attentional training in patients who underwent CABG compared to patients who underwent CABG and received no additional cognitive training.

The purpose and primary outcome of this prospective, randomized and controlled study is to evaluate the effectiveness of a paper-and-pencil-based cognitive training program on objectively measured cognitive functions for patients undergoing cardiac surgery with extracorporeal circulation. The primary outcome will be evaluated directly after training at discharge from rehabilitation. The delivery of the cognitive training will start at the beginning of rehabilitation, which means approximately 1 week after surgery. Secondary outcomes are the training effect on cognitive functions, depression and health-related quality of life 3 and 12 months after discharge from rehabilitation, and the impact of perioperative cerebral ischemia on the training effect. Perioperative cerebral ischemia will be measured with postoperative diffusion-weighted magnetic resonance imaging (DW-MRI) during the first postoperative week. To our knowledge, this is the first study to explore the impact of cerebral ischemia on cognitive training for patients who have undergone cardiac surgery.

## Methods

### Study design and enrollment

This study is a multicenter, randomized controlled trial conducted at the department of cardiac surgery of the Kerckhoff Heart and Thorax Center in Bad Nauheim, Germany, and at the department of cardiovascular surgery of the University Hospital Giessen, Germany. It complies with the Declaration of Helsinki and has been approved by the ethics committee of the Justus Liebig University Giessen (ref. 28/14). The study coordinator receives information about planned elective cardiac surgery from the participating study centers, which he screens according to eligibility criteria. Potential patients will be contacted and informed verbally and in writing in detail about the purpose, procedure, and possible consequences of the study project. If the patient agrees to participate, a written informed consent form will be signed by the patient and the investigator prior to the patient’s enrollment.

Our study team consists of members of the departments of neurology, neuropsychology, neuroradiology, heart surgery, and rehabilitation who are responsible for running the study, including preparing the protocol, monitoring the study and writing the study reports. The study protocol follows the SPIRIT (Standard Protocol Items: Recommendations for Interventional Trials) guideline (see Additional file 1).

Due to the planned small sample size, the expected lack of harm and the relatively short execution time of the cognitive training (3 weeks), the implementation of a data monitoring committee was not considered.

All patients will pass a detailed neuropsychological assessment the day before surgery, at discharge from rehabilitation, and at 3 and 12 months after discharge. At every time point, patients will complete a standardized questionnaire on depression and anxiety. Questions about cognitive failure in daily life and HQL will be assessed before heart surgery and at 3 and 12 months after discharge from rehabilitation. Before surgery, data documentation will include age, sex, education, body mass index, preexisting conditions, and medication. Documentation of perioperative date will be the type and amount of anesthesia administered, duration of anesthesia administration, type and amount of analgesia administered, duration of analgesia administration, duration of surgery, duration of extracorporeal circulation, cross-clamp time and perioperative complications. Post-surgery intensive care unit (ICU) days, total length of inpatient stay, postoperative complications and postoperative delirium will be recorded. At 6–10 days after surgery, MRI of the brain will be conducted to screen for cerebral ischemia, hemorrhage, or other acute pathologic conditions potentially confounding neuropsychological assessment and the effects of cognitive training. Following the inpatient stay in the acute hospital (approximately 7 days), patients will be directly transferred to the department of rehabilitation at the Kerckhoff Clinic in Bad Nauheim, Germany.

**Fig. 1:**
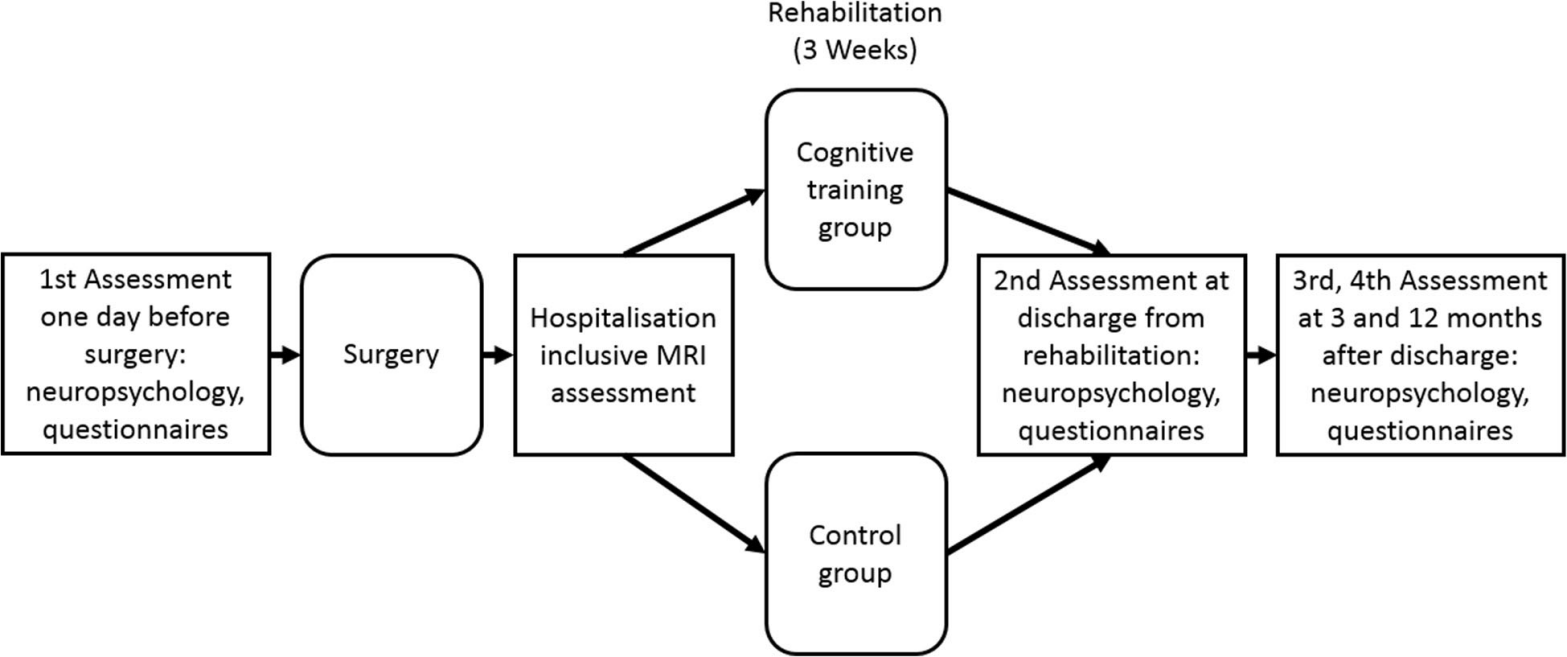
Timeline of study-related events. The first assessment includes neuropsychological tests and questionnaires on the day before surgery. Within 6–10 days after surgery, magnetic resonance imaging (MRI) of the brain is scheduled. After the acute hospitalization, patients will be transferred to the center of rehabilitation and randomly assigned to the cognitive training group or the control group. A second neuropsychological assessment will be administered at discharge from rehabilitation. To determine the long-term effects of the cognitive training, a neuropsychological test and questionnaires will be administered at 3 and 12 months after discharge from rehabilitation

During their stay at the rehabilitation center, which usually lasts 3 weeks, all patients will receive standard cardiac rehabilitation including physical exercise, medical management and nutritional counseling. The cognitive training group will undergo an additional cognitive training program consisting of paper-and-pencil exercises. The study design is shown in Fig. 1. A detailed trial schedule in accordance with the Standard Protocol Items: Recommendation for Interventional Trials (SPIRIT) guideline is shown in Table 1.

**Table 1:**
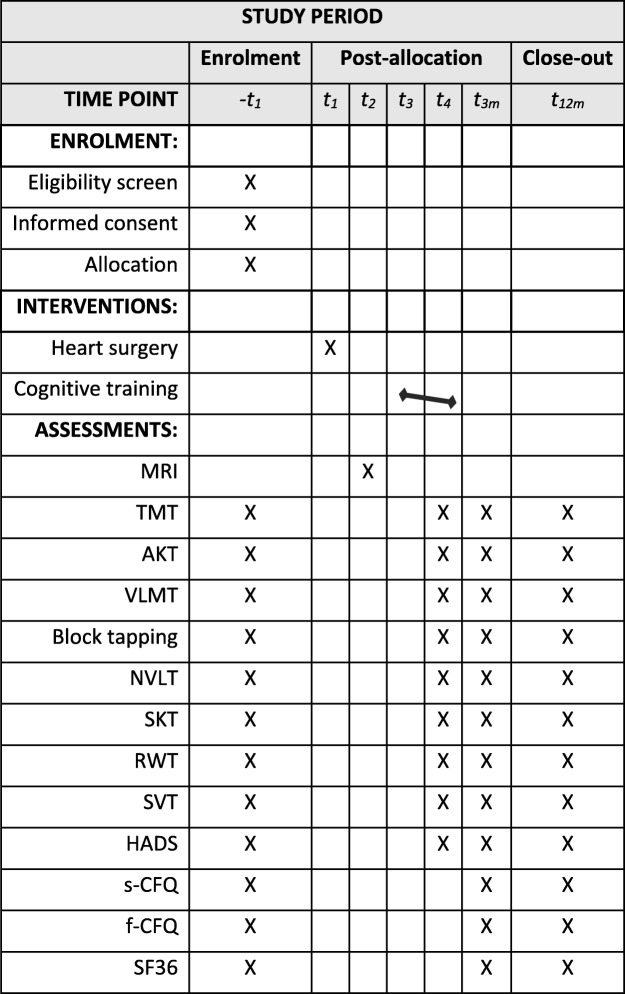
Trial schedule of enrollment, interventions, and assessments

*MRI* magnetic resonance imaging, *TMT* Trail Making Test, *AKT Alterskonzentrationstest*, *VLMT Verbaler Lern- und Merkfähigkeitstest*, *NVLT* Non-Verbal Learning Test, *SKT Syndrom-Kurztest*, *RWT Regensburger Wortflüssigkeits-Test*, *SVT Symbolverarbeitungstest*, *HADS* Hospital Anxiety and Depression Scale, *s-CFQ* Cognitive Failure Questionnaire for self-assessment, *f-CFQ* Cognitive Failure Questionnaire for foreign assessment, *SF36* Short Form-36

### Inclusion and exclusion criteria

Patients receiving elective aortic or mitral valve replacement/reconstruction with or without CABG will be included in this study. All heart operations will be performed with standard extracorporeal circulation. Due to the use of a standardized psychological assessment, patients must be native German speakers. Exclusion criteria are history of stroke and preexisting psychiatric or neurological disorders. Patients whose health insurance does not grant the postoperative treatment in the department of rehabilitation at the Kerckhoff Clinic in Bad Nauheim (Germany) must also be excluded.

If patients no longer wish to participate in the cognitive training or neuropsychological examination due to a deteriorating state of health, lack of motivation, any other reason, or without reasons given, they may discontinue participation in the study. Furthermore, participants will be excluded from the study if they are transferred to another clinic during their stay in the rehabilitation center.

Medical and psychological interventions in the context of other studies that may exert effects on patients’ cognition are prohibited. In general, concomitant care and interventions as part of the standard rehabilitation program are permitted.

### Randomization

After enrollment and a baseline assessment, patients will randomly be assigned by the study coordinator to the cognitive training group or the control group, which will not receive cognitive training. Patients will be randomized using a computer-generated randomization list with a 1:1 blocked allocation ratio. The randomization list will be sequentially numbered and will be generated by the study coordinator prior to the start of the study.

### Blinding

Surgeons, radiologists and neuropsychologists who are involved in the outcome variables will be blinded to the randomization status. Cognitive testing and training will be carried out by two different, experienced neuropsychologists in order to maintain the blinding. During follow-up assessments, patients may tell the blinded neuropsychologist accidentally before the start of the neuropsychological test whether or not they have received previous cognitive training. In this case, however, the neuropsychological test will be performed and discussed in the study reports.

### Neuropsychological assessment

A battery of cognitive tests will be performed by a neuropsychologist on the day before surgery, at discharge from rehabilitation, and at 3 and 12 months after discharge. When available, parallel test forms will be used at follow up to account for learning effects. The order in which the parallel test forms are presented will be counterbalanced so that each parallel test form occurs with the same frequency at each test time point.

The cognitive test battery assesses selective attention, verbal and visual memory with short-delay and long-delay episodic memory conditions, verbal working memory, cognitive flexibility, word fluency and symbol processing. Selective attention will be examined using the Trail Making Test A (TMT-A) [20] and the *Alterskonzentrationstest* (AKT) [21]. In the TMT-A, the patient has to link numbers in ascending order on a test sheet. The AKT consists of a matrix of similar visual stimuli, where a target stimulus has to be marked.

To assess verbal memory, the *Verbaler Lern- und Merkfähigkeitstest* (VLMT), a modified German version of the Rey Auditory Verbal Learning Test [22], will be applied. This test can be used to evaluate short-term memory, learning, episodic memory, and verbal discriminability. First, the patient has to concentrate on a word list that is read out loud by the investigator. The direct retrieval of the patient is scored as short-term memory performance. Second, the patient has to learn the word list in five learning trials. The sum of the recalled words represents a learning parameter. Third, a second word list with new words is presented verbally only once, to divert attention from the first word list. After this, the learned words of the first word list have to be recalled; this is used as a measurement of a short-delayed function of verbal episodic memory. A second verbal episodic memory measurement is performed 20 min later (long delay). Finally, the verbal recognition ability is proven by discriminating between already learned and new words. Between the short-delayed verbal episodic memory trial and the long-delayed verbal episodic memory trial, nonverbal cognitive tests are performed to avoid the potential effect of interfering words not included in the learned wordlist.

Visual memory will be examined using the Block-Tapping Test [23], the Non-Verbal Learning Test (NVLT) [24] and the pictorial memory subtest of the German *Syndrom-Kurztest* (SKT) [25]. In the Block-Tapping Test, the patient has to tap blocks on a board with his or her hand in a given order forward and backward. The NVLT is a test to evaluate the visual recognition of repeated abstract symbols within a variety of 60 cards. The pictorial memory subtest of the German SKT will be administered to the patient to evaluate short-term episodic memory and recognition of 12 visual pictures, which are presented in one learning trial. With the Letter Number Test, a subtest of the MATRIX test battery, the verbal working memory is tested through the mental reorganization of numbers and letters [26]. Cognitive flexibility will be assessed by the Trail Making Test B (TMT-B) [20], where numbers and letters have to be alternately linked, and by another subtest of the SKT (SKT 7) [25], where the patient has to name interfering letters (e.g., “A” instead of “B,” and vice versa).

Furthermore, semantic and phonetic verbal fluency will be tested using the “Regensburger Wortflüssigkeits-Test” (RWT) [27]. In this test, in 1 min, the patient has to name words from a specific category to test semantic fluency and words with a specific initial letter to test phonetic fluency. At the end of the test battery, the *Symbolverarbeitungstest* (SVT) will be performed to test the processing of symbolic pictures [28].

### Questionnaires

Study patients will complete a validated German version of the Cognitive Failures Questionnaire for self-assessment (s-CFQ) [29]. Close relatives of the patients will answer a cognitive questionnaire to evaluate foreign assessment (f-CFQ) [30]. The questionnaires will examine the frequency of failures in daily living related to memory, attention, action, and perception. Because memory impairment is an important element that affects everyday functioning, the s-CFQ was modified with additional questions related to memory failures, taken from the validated German version of the Memory Complaint Questionnaire [31]. Depression and anxiety will be scored using the validated German version of the Hospital Anxiety and Depression Scale (HADS) [32]. HQL will be assessed using the Short Form-36 (SF36) questionnaire [33]. The SF36 covers eight health-related factors including vitality, physical functioning, bodily pain, general health perceptions, physical role functioning, emotional role functioning, social role functioning, and mental health. The HADS will be used at every neuropsychological test time point; the s-CFQ, f-CFQ and SF36 will be completed at baseline and 3 and 12 months after discharge from rehabilitation.

### Magnetic resonance imaging

Cranial MRI will be performed 6–10 days after surgery using a 3-T scanner (Skyra; Siemens, Erlangen, Germany). The protocol of imaging will include a T2-weighted turbo spin-echo sequence (slice thickness = 3 mm, field of view (FOV) = 220 × 220 mm, matrix = 512 × 391, repetition time (TR) = 7490 ms, echo time (TE) = 100 ms), a T2-weighted turbo spin-echo sequence for dark fluid (slice thickness = 3 mm, FOV = 220 × 220 mm, matrix = 320 × 224, TR = 7000 ms, TE = 81 ms), a T1-weighted FLASH sequence (slice thickness = 3 mm, FOV = 220 × 220 mm, matrix = 320 × 320, TR = 250 ms, TE = 2.49 ms) and a diffusion-weighted echo-planar imaging sequence (slice thickness = 3 mm, FOV = 220 × 220 mm, matrix = 160 × 160, TR = 7720 ms, TE = 64 ms, slice gradients of *b* values = 0 and 1000 s/mm^2^). The postoperative diffusion-weighted sequence will be used by two blinded, experienced observers for registration and planimetric evaluation of acute ischemic lesions.

### Primary outcome measure

The primary outcome measure will be the training effect on all objectively measured neuropsychological functions at discharge from rehabilitation.

### Secondary outcome measure

As a secondary outcome, we will evaluate the training effect of all objectively measured neuropsychological functions at 3 and 12 months after discharge from rehabilitation. Second, we will evaluate the training effect on the subjective self-assessment and external assessment by relatives of cognitive failures at 3 and 12 months after discharge from rehabilitation. Third, we will evaluate the training effect on HRQ at 3 and 12 months after discharge from rehabilitation. Fourth, we want to investigate the extent to which the cognitive training has an impact on depression at all follow-up time points. Last, we will examine the impact of perioperative cerebral ischemia on the neuropsychological measured training effect at all follow-up time points. Perioperative cerebral ischemia will be measured with postoperative DW-MRI during the first postoperative week. The number and size of ischemic lesions are determined and will be used as a control variable for the analysis of the training effect.

### Cognitive training

There is currently only one study in which effective cognitive training was performed in patients undergoing cardiac surgery [19]. This study used a combination of computer-based training (with a focus on selective attention) and memory strategy training (method of loci). We decided against this training concept because we think that our elderly patients are not familiar with the use of computers, and we will therefore use a purely paper-and-pencil approach. Second, memory strategies seem to be less effective than cognitive exercises such as computerized or paper-and-pencil procedures [34]. To our knowledge, there is no specific cognitive domain that clearly emerges over time in the context of POCD, such as memory or attention. Therefore, we decided to train several cognitive functions that are used especially in everyday life to maintain social functions and earning capacity. These include word fluency, working memory, attention, and the ability to plan.

For the preparation of our training program we first conducted a literature search on German-language-validated paper-and-pencil-based cognitive exercise methods. The literature is very scarce. In a controlled study design, a training program by Müller et al. (2004) [35] showed cognitive improvements in patients with executive dysfunction [36]. Their program included training in word fluency, cognitive flexibility, working memory, and planning ability. However, we found the cognitive training of Müller et al. (2004) [35] in some parts to be too unentertaining for our patients, whereby we took over only a few training tasks and combined them with new tasks designed by our group to achieve better acceptance.

Cognitive training in the intervention group will include paper-and-pencil-based exercises practicing multi-domain cognitive executive functions such as word fluency, verbal and visual working memory, selective visual attention, and planning. One training session will last approximately 40 min and will be performed 6 days a week for 3 weeks.

The daily training program consists of eight different types of standardized tasks addressing the processing of words, categories, images, head calculation, and planning. New words, categories, images, head calculations, and planning tasks will be presented on each training day. Each task takes between 2 and 10 min; to manage the working time, the patients must limit their work using a digital clock. At the beginning of the training program, a trained investigator will give explicit instructions in a one-to-one training session and will be nearby to help with any questions about the exercises. If no further help is needed in the following training days, the patient will be provided with training material for the following 6 days so that patients can complete the training independently in their ward rooms. Each task contains precise written instructions that can be used to assist in its execution. If a patient has questions about the training, he or she can contact the trainer. After every 6th day, the extent to which the tasks have been completed is checked by the training investigator and new training material will be provided. Patients are told that their exercise solutions are not evaluated or corrected. Therefore, it does not matter whether the solutions are right or wrong. An important concern for the patients is that they concentrate on the tasks and cognitively exert themselves. In this way, we can avoid possible performance pressure and also avoid patients exchanging the right approaches among themselves. The different types of tasks are presented in the following standardized order.

#### Phonetic word fluency

The patient is given three letters on a sheet of paper. Within 2 min, he or she has to note as many words as possible that begin with these letters. This task is mainly intended to train word fluency and was adapted from Müller et al. (2004) [35].

#### Categorical word fluency

In this task, the patient is given three different categorical terms on a sheet of paper. Within 2 min, as many words as possible that can be assigned to these categories must be found and noted. This task is mainly intended to train word fluency and was adapted from Müller et al. (2004) [35].

#### Comic strips

Patients receive 4–5 popular German comics from German illustrators such as Wilhelm Busch, Erich Ochser or Hans Jürgen Press, with 3–16 pictures of a story in mixed order. Within 5 min, the pictures have to be arranged mentally in a meaningful order. The new invented order should be documented by numbering the pictures with a pencil. This task is mainly intended to train working memory and was created by our group.

#### Mental arithmetic

The patient is asked to complete several calculation tasks on a sheet of paper. The result of the first arithmetic problem, which includes addition, subtraction and multiplication of numbers, must be memorized. In the second step, another calculation task must be solved, and the result must also be memorized. In the last step, the last result should be subtracted from the first result, and the final result should be written down. The time limit for this exercise is 5 min. This task is mainly intended to train working memory and was adapted from Müller et al. (2004) [35].

#### Synonymic fluency

The next worksheet contains three different terms. For each term, patients must find words with similar meanings (synonyms). For example, if the term is *wallet*, then other words would be *portmonee* or *money purse*. The time limit is 2 min. This task is mainly intended to train word fluency and was created by our group.

#### Fill in the blank text

In the next training task, short stories are presented. These are generally known stories by Wilhelm Busch, the Brothers Grimm, Hans Christian Andersen or fables from antiquity, German studies, Buddhism, and Japan. The stories have gaps that have to be filled in with a self-chosen, meaningful word. The time limit for this exercise is 5 min. This task is mainly intended to train word fluency and working memory and was constructed by our group.

#### Where is Waldo

An illustration of Martin Handford’s “Where is Waldo?” is presented on a DIN A3 sheet of paper. The picture contains dozens or more people doing a variety of things in a particular place. The patient has to find some specific signs or objects listed on a sheet of paper by marking them with a pen on the DIN A3 sheet within 5 min. This task is mainly intended to train selective attention and working memory and was created by our group.

#### Planning

In the last task, the patient must read a text in which an imaginary person has to perform transactions or organize appointments. The patient’s task is to solve the problems by writing down a concrete solution. The time limit for this task is 10 min. The task is mainly intended to train planning ability and working memory and was adapted from Müller et al. (2013) [37].

### Planned statistical analyses

The effect of cognitive training will be analyzed by repeated measures (mixed between-within) analysis of variance (ANOVA) with groups (control group/intervention group) as the between-subject factor and assessment time (baseline and all follow-up assessments) as the within-subject factor for all cognitive tests and questionnaires, respectively. Assumptions for repeated measures ANOVA will be tested using the Levene test for variance-homogeneity and Mauchly’s test for sphericity. If sphericity is violated, alpha levels will be adjusted using the Greenhouse-Geisser correction. Normal distribution will be tested using the Shapiro-Wilk test. To control for the possibility of confounder variables that could affect the results, we will conduct additional repeated measures analysis of covariance (ANCOVA), which includes covariates such as preoperative cognitive values, age, sex, education, psychiatric scores (depression/anxiety), and perioperative variables such as duration of extracorporeal circulation, administration of anesthesia, and number and size of ischemic lesions.

Post hoc explorative between-subject comparisons will be analyzed using the *t* test for independent samples. In this case, we will compare the intervention group with the control group by calculating a change score in cognitive values (post-training score minus pre-training score). The change in cognitive values is the dependent variable, and the treatment group (cognitive training group/control group) is the independent variable.

Within-subject comparisons will be analyzed using the paired *t* test. When the assumptions for parametric tests (normal distribution, variance-homogeneity between two groups) are not given, the variables will be analyzed using non-parametric variance techniques. In this case, between-group differences will be calculated using the Mann-Whitney U test, using change scores in cognitive values (post-training score minus pre-training score) and the Wilcoxon signed-rank test for within-group comparisons. Nominal variables will be analyzed by Pearson’s chi-squared test. Depending on the parametric level of the data, correlation with continuous variables will be calculated using Pearson product-moment correlation, Spearman rank correlation, or Kendall tau correlation. Effect size of the cognitive training will be calculated by the difference in the pretest-posttest measure between the intervention and control group, weighted by the pooled standard deviation of the pretest measurement, because this is the recommended choice for a pretest-posttest controlled design [38]. The criterion for statistical significance will be set at *p* < 0.05. In the case of multiple tests, we will control *p* values using the false discovery rate (FDR) correction method [39]. Since we expect dropout of some patients on follow-up assessments, all patients will be included in the intention-to-treat analysis, where missing data will be imputed by a multiple imputation method. To evaluate the impact of missing data, a complete case analysis will also be performed, followed by best-worst and worst-best case sensitivity analyses.

Another approach to identifying a training effect on cognition will be to compare the frequencies of POCD and POCI between the groups using Pearson’s chi-squared test. POCD will be defined as a decline from pre to post assessment of 1 standard deviation in at least two neuropsychological parameters as this is a widely used method [2]. Similarly, POCI will be defined as an improvement from pre to post assessment of 1 standard deviation in at least two neuropsychological parameters.

Interim analyses will be carried out during the study period to identify adverse events, an overwhelming effect, or the futility of the experimental arm. In this case, the study could be terminated prior to its completion. The decision will be made by the study team.

### Power and sample size estimation

Cognitive training for patients undergoing heart surgery administered by de Tournay-Jette et al. (2012) [19] revealed medium-to-large effect sizes (*η*^2^ = 0.10–0.23). We have decided to use the smaller effect size (*η*^2^ = 0.10) for calculation so as not to underestimate the required sample size. Using this effect size, 37 patients per group are needed to obtain statistical power of 80% at a significance level of *p* = 0.05 (two-tailed). Based on previous cardiosurgical studies, we estimate a dropout rate of 20% between each of the four assessment time points. Thus, the number of study patients recruited at baseline assessment was fixed at 72 patients per group. For sample size and statistical power calculations, we used G*Power-3 analysis software.

### Data management

All personal information about enrolled patients will be subject to medical confidentiality. Paper-based assessment forms will be used to record the outcome variables. The data will be manually entered in an electronic database, which is password-protected and will be checked for quality and accuracy. All assessment forms, signed informed consent forms, and the randomization list will be stored in a locked cabinet.

### Dissemination policy

Our goal is to make the study results available to the general public, healthcare providers, and scientists by publishing them in the public press, at scientific congresses, and as original articles in peer-reviewed journals. The results will be reported regardless of the amount and direction of the effect.

## Discussion

The aim of this study is to evaluate a paper-and-pencil-based cognitive training program for patients undergoing heart surgery with extracorporeal circulation. POCD shows the highest incidence of neurological complications that can occur in the context of surgery, especially cardiac surgery [1]. Adverse effects of POCD on daily living abilities can be noticed by patients themselves and their relatives in both the short and long term [6]. The etiology remains controversial but can be considered a multifactorial event in which extracorporeal circulation plays a crucial role [8].

Here, we present cognitive training that integrates well with standard rehabilitation, takes place as early as possible, and aims to reduce the occurrence and persistence of cognitive deficits in the short and long term. It is also of interest whether patients with perioperative ischemic stroke benefit from this intervention to varying degrees or in different forms.

We intentionally opted for technically simple paper-and-pencil-based training tasks because POCD after cardiosurgical procedures mainly affects elderly people. Many studies on the effect of cognitive training have used computer-based training tasks [15], which provide the advantage of generating and capturing data simply, quickly and very precisely. However, even today, they often represent an unfamiliar medium for older people, which could lead to irritation, fear of contact, and/or frustration in the older population studied here and thus constitute a potential bias. A placebo intervention for the control group is intentionally omitted because cognitive effects of placebo interventions on cognitive performance are difficult to control. In order to credibly suggest to patients that the placebo intervention could have an influence on their memory and thus also to achieve a willingness to participate, the structure of the placebo intervention will have to be closely related to cognitive training (e.g., relaxation exercises, crossword puzzles, conversation therapy, computer games, etc.) and thus also achieve cognitive training effects.

The only prospective investigation of patients undergoing cardiac surgery to date reported a beneficial effect of memory and attentional training in patients who underwent CABG [19], but no information was given on the use of extracorporeal circulation in this collective. In this study, training took place between the 6th and the 10th week following surgery. However, it is still not clear when the optimal time point for cognitive training should be set and for how long the intervention should be carried out. It seems possible that the rehabilitation of postoperatively impaired cognitive function will be more improved by an earlier intervention starting in the range of a week compared to one month after surgery. Controlled studies on neurorehabilitation of post-stroke cognitive impairment have shown beneficial effects for several cognitive functions when restitutive cognitive training begins within 2 weeks [40] or about 7 months [17] after stroke onset. Animal models suggest a time-dependent increase in neuroplasticity (plastic window) after ischemic injury, with a peak at 7–14 days and near completion at 30 days. Nevertheless, it should be noted that the assignability of these findings from bench to bedside is difficult, and the shape and extent of the neuroplastic window in humans remains unclear [41].

In our study, the detection of acute, perioperative cerebral ischemia is performed by MRI with DWI. With the use of DWI, a preoperative baseline assessment is not necessary because in this sequence, acute ischemic tissue is presented as a bright area that typically occurs 2–3 h after the onset of damage and usually subsides within 2–3 weeks [42]. These acute cerebral ischemic lesions can be detected by DW-MRI in 14–61% of the patients after cardiac surgery [11, 43]. The significance of those acute MRI lesions, especially with regard to manifestation of POCD, remains unclear [42]. To our knowledge, there are no studies controlling for the potentially confounding effect of peri-interventionally endured cerebral ischemia detected during the first postoperative week. In this context, it seems of particular interest whether patients with acute cerebral ischemia benefit from cognitive training to any other degree or in any other form.

So far, increased efforts have been made to prevent POCD, addressing anesthesia, cardiotechnology, and cardiac surgery [9], with limited but measurable success; these procedural preventive measures alone can reduce the incidence of POCD in the magnitude of 30% [44]. In contrast to procedural prevention strategies, the concept of cognitive training allows the patient to act independently, responsibly and actively. This can perhaps exert a positive effect on the recovery process through the experienced self-efficacy that seems to contribute to better recovery after heart surgery in terms of patients’ worries, energetic mood, reading, ambulating, and fitness [45]. With the knowledge that cognitive deficits can be actively addressed afterward, the fear of surgery can possibly be reduced; finally, one must not forget the drastic experience of a potentially life-threatening cardiac disease. Preoperative anxiety has been shown to be a predictor of major morbidity and mortality in patients who have undergone heart surgery [46].

The present investigation certainly contains some limitations. First of all, participation in the study is tied to the performance of a heart operation using a heart-lung machine. A comparison with patients undergoing off-pump cardiac surgery is therefore not possible. Given the lack of knowledge about the optimal starting time or duration of training, the intervention may be too early or the duration of approximately 3 weeks too short. It could also be discussed as to whether an additional follow-up investigation after several years would provide further valuable information on the long-term course of POCD and the training effects. For the aforementioned reasons, no placebo intervention is planned in the present study; this may formally affect the quality of the study and the transferability of the results. Finally, our cognitive training consists of a combination of validated [36] and unvalidated tasks. Since our self-created tasks are not validated, we do not know whether they might potentially contribute to an alteration in cognition.

The results of our study could have potentially important implications for the prevention and treatment of POCD. In particular, if our cognitive training is feasible and effective in maintaining or improving cognition and quality of life, it could be integrated into the treatment programs of rehabilitation centers that treat patients after heart surgery. Economically and organizationally, this is possible without much effort because the cognitive exercises are independently workable and need no great control, and rehabilitative centers usually have psychologists who could be involved in the organization of the exercises, distribution of tasks, or clarification of questions. Furthermore, the cognitive training is designed in such a way that it could also be carried out in an ambulant setting.

### Trial status

The study is currently enrolling patients. Recruitment started on 13 July 2016. Recruitment is expected to be completed in February 2020. Protocol version: 1.4 (10-08-2019).

## Supplementary information

**Additional file 1.** SPIRIT 2013 Checklist: recommended items to address in a clinical trial protocol and related documents.

## Data Availability

The datasets analyzed during the current study are available from the corresponding author on reasonable request.

## Abbreviations

AKT: Alterskonzentrationstest
ANCOVA: Analysis of covariance
ANOVA: Analysis of variance
CABG: Coronary artery bypass grafting
DW-MRI: Diffusion-weighted magnetic resonance imaging
FA: Fractional anisotropy
f-CFQ: Cognitive Failure Questionnaire for foreign assessment
FDR: False discovery rate
FOV: Field of view
HADS: Hospital Anxiety and Depression Scale
HQL: Health-related quality of life
ICU: Intensive care unit
MCI: Mild cognitive impairment
MRI: Magnetic resonance imaging
NVLT: Non-Verbal Learning Test
POCD: Postoperative cognitive decline
POCI: Postoperative cognitive improvement
RWT: Regensburger Wortflüssigkeits-Test
s-CFQ: Cognitive Failure Questionnaire for self-assessment
SKT: Syndrom-Kurztest
SVT: Symbolverarbeitungstest
TE: Echo time
TMT: Trail Making Test
TR: Repetition time
VLMT: Verbaler Lern- und Merkfähigkeitstest
